# Results of Lung Cancer Screening in a Rural Setting: A Retrospective Cohort Study

**DOI:** 10.7759/cureus.23299

**Published:** 2022-03-18

**Authors:** Bridger Bodily, John Ashurst, Jason Fredriksen, Brent Bedke, Adam Braze, Robert Matheny, Jay Vlaminck

**Affiliations:** 1 Graduate Medical Education, Kingman Regional Medical Center, Kingman, USA; 2 Emergency Medicine, Kingman Regional Medical Center, Kingman, USA; 3 Radiology, Kingman Regional Medical Center, Kingman, USA; 4 Pathology, Kingman Regional Medical Center, Kingman, USA; 5 Surgery, Kingman Regional Medical Center, Kingman, USA; 6 Medicine, Kingman Regional Medical Center, Kingman, USA

**Keywords:** low-dose computed tomography, cancer, community health research, lung cancer screening, lung cancer

## Abstract

Background

In this study, we aimed to determine the performance of the lung cancer screening (LCS) program using low-dose computed tomography (LDCT) in a rural community.

Methodology

We conducted a retrospective cohort study of patients who underwent LCS at a rural healthcare institution from September 1, 2016, through December 31, 2019, to determine the utilization of screening, rate of positivity, rate of cancer detection, and patient compliance.

Results

A total of 1,474 patients underwent initial LCS, and 1,776 LCS examinations were performed using LDCT. Of 1,776 tests performed, 375 (21.1%) were categorized as positive (Lung CT Screening Reporting and Data System III or higher), with 215 of the 375 (57.6%) being lost to follow-up. A total of 29 malignancies were identified (in 1.6% of all LCS tests) during the study period, with 23 (82.8%) malignancies being low-stage malignancies (stage I or II), 24 (79.3%) malignancies potentially surgical candidates (stage IIIA or less), and five (17.2%) malignancies being non-surgical candidates based on stage (stage IIIB or IV). A total of 28.7% of all patients eligible for repeat screening had at least one repeat annual test. Overall, 9.9% of all patients eligible for two repeat annual tests had a second repeat annual test.

Conclusions

LCS using LDCT is effective in detecting lung cancer in a rural setting. However, compliance with repeat annual screening and recommendations for further workup is low. This may be exacerbated by healthcare and socioeconomic issues prevalent in rural communities. The use of LCS patient coordinators and dedicated tracking software may improve compliance with repeat annual screening and compliance with recommendations when LCS tests are positive.

## Introduction

Lung cancer is the leading cause of cancer death in the United States and is primarily caused by tobacco-related diseases [1,2]. Detecting lung cancer at an early stage has been a long-held collective goal of public healthcare institutions and may allow potentially curative treatment or increased odds of long-term survival [3,4]. This need is even more pressing in rural communities because tobacco use rates are higher in rural communities relative to urban areas [5].

The United States National Lung Screening Trial (NLST) showed a 20% decrease in lung cancer-specific mortality in a high-risk population using low-dose computed tomography (LDCT) [6]. The cost-effectiveness of LDCT lung cancer screening (LCS) has been established [7]. Since 2011, most professional organizations have recommended LCS with this methodology, and guidelines for screening programs have been published [3]. In 2014, the US Preventative Services Task Force recommended LCS [8]. On February 5, 2015, the Centers for Medicare and Medicaid Services (Medicare) issued its final decision about coverage of LDCT for LCS [9].

The NLST stated that one weakness of the study was that the trial was conducted at institutions “which are recognized for their expertise in radiology and the diagnosis and treatment of cancer. It is possible that community facilities will be less prepared to undertake screening programs” [6]. Furthermore, community facilities in smaller rural settings often face greater limitations in resources and expertise [10-13]. To evaluate these concerns, we sought to describe the performance of an LCS program within a non-NLST rural community hospital.

## Materials and methods

Setting

Kingman Regional Medical Center is a 235-bed rural community hospital in northern Arizona with an annual emergency department census of approximately 55,000 patient visits. The hospital serves District 1 of Mohave County and covers an approximate 10,000 square-mile area.

Methods

The study was approved (KHI 0193) by the Kingman Healthcare Incorporated Institutional Review Board (IRB) and patient consent was waived due to the retrospective nature of the study. Following IRB approval, we conducted a retrospective chart review of all patients who underwent LCS with LDCT from September 1, 2016, through December 31, 2019. LCS was performed using the Centers for Medicare & Medicaid Services eligibility criteria (age 55-77 years, at least 30-pack-year smoking history, smoking cessation less than 15 years, no cancer symptoms, no concomitant life-threatening illness, medically fit, and willing to undergo additional indicated medical management and future LCS), and examination techniques were performed following the American College of Radiology standard imaging protocols [14]. Screening tests were interpreted by board-certified radiologists using a standard structured reporting and the Lung CT Screening Reporting and Data System (Lung-RADS) protocol for lung nodule evaluation and subsequent recommendations. Primary care clinicians managed subsequent visits following the initial LCS until referral to specialists. All data were abstracted from the hospital’s LCS program registry using a structured abstraction tool after initial abstractor training. Abstracted data included the number of patients screened, number of patients who returned for repeat screening, results of LDCT tests, number of findings in each of the Lung-RADS categories, number of patients subsequently diagnosed with lung cancer, and stages of lung cancer in those patients.

Statistical analysis

Statistical analysis was conducted using Microsoft Excel. Nominal data are presented as descriptive statistics. 

## Results

A total of 1,474 patients underwent initial LCS, and a total of 1,776 LCS examinations were performed using LDCT. All LCS examinations were assigned a final Lung-RADS score using the American College of Radiology diagnostic criteria (Table 1). A total of 968 patients were eligible for one or more repeat annual LCS tests by having their initial test completed by December 2018, and 28.7% (268/968) of the eligible patients had at least one repeat annual LCS test. A total of 537 patients were eligible for two repeat annual LCS tests by having their initial test completed by December 2017, with 9.9% (53/537) having a second repeat annual test.

**Table 1:** The Lung-RADS scoring system. Lung-RADS: Lung CT Screening Reporting and Data System

Lung-RADS score	Findings
1 – Negative	No nodules and definitely benign nodules
2 – Benign appearance or behavior	Nodules with a very low likelihood of becoming a clinically active cancer
3 – Probably benign	Nodules with a low likelihood of becoming a clinically active cancer
4A – Suspicious	Findings for which additional diagnostic testing is recommended
4B – Very suspicious	Findings for which additional diagnostic testing and/or biopsy is recommended

The average age of this cohort was 66.1 years. Of the 1,776 tests, 931 (52.4%) were performed on female patients and 845 (47.6%) were performed on male patients. Of the 1,776 tests performed, 375 (21.1%) were positive with Lung-RADS scores of 3, 4A, 4B, or 4x (Table 2). Of the 375 positive LCS tests, 27.9% (104/375) were determined to be benign by diagnostic positron emission tomography scan. A total of 14.5% (54/375) of the positive tests underwent biopsy. No evidence of further workup was available in 57.6% (215/375) patients with positive tests.

**Table 2:** The Lung-RADS score of those who underwent lung cancer screening with low-dose computed tomography from September 1, 2016, through December 31, 2019. Lung-RADS: Lung CT Screening Reporting and Data System

Lung-RADS score	Total cases (N = 375)
RAD 3	189 (50.7%)
RAD 4A	95 (25.5%)
RAD 4B	84 (22.5%)
RAD 5	5 (1.3%)

In the total cohort, malignancies were identified in 2% (29/1474) of all patients screened (Table 3). Of the 1,776 LCS tests performed, lung cancer was identified in 1.6% of total LCS tests performed. A total of 82.8% (23/29) malignancies were low-stage malignancies (stage I or II), 79.3% (24/29) were potentially surgical candidates (stage IIIA or less), and 17.2% (5/29) were not surgical candidates based on stage (stage IIIB or IV). The average number of patients needed to screen to detect one lung cancer of any stage was 61 tests. The number needed to screen to detect one lung cancer of a stage that was potentially surgically resectable was 74 tests. The number needed to screen to detect one lung cancer of a low stage was 77 tests.

**Table 3:** Malignancy staging of those who underwent lung cancer screening with low-dose computed tomography.

Stage	Total cases (N = 29)
I	17 (58.6%)
II	6 (20.6%)
IIIA	1 (3.4%)
IIIB	2 (6.9%)
IV	3 (10.3%)

## Discussion

Since the completion of the NLST, LCS by LDCT scans has become encouraged and supported by healthcare and patient advocacy organizations nationwide [3,8,9]. The NLST was a large-scale trial that analyzed over 75,000 LCS tests using LDCT, with 24.2% of all screening tests being classified as positive, and 1.4% of all screening tests resulting in a cancer diagnosis [6]. The current study found similar results, with 21.1% of all total screening tests being classified as positive, and 1.6% of all total screening tests resulting in a cancer diagnosis. This data suggest that LCS with LDCT is effective at detecting lung cancer in a rural patient population represented by our LCS Program at Kingman Regional Medical Center.

One concerning finding from the study is that 57.6% of patients with positive LCS tests did not pursue further testing or treatment at Kingman Regional Medical Center. It is possible that a proportion of these patients did pursue further care at a larger institution due to the perceived benefit of a further workup at an academic institution or a larger institution due to the potential seriousness of the diagnosis.

There is currently no way to definitively calculate the rate of initial compliance with LCS in the rural community because there are no definitive data on the number of patients eligible to undergo LCS. To estimate the compliance with LCS, available census and county health data reported that approximately 24% of the local population was between the ages of 55 and 77 years, and the active smoking rate among all individuals in the county was 29% [15]. If it is estimated that one-third of smokers between the ages of 55 and 77 in Mohave County have a smoking history of at least 30-pack-years and continue to smoke or ceased to quit smoking less than 15 years ago, then 1,630 people in the catchment area could have potentially qualified for the annual LCS program. Given that 1,473 patients received at least one LCS test over the time period studied, it is likely that the LCS rate for the initial LCS test is relatively high (90%), but the proportion of possible patients receiving the test annually is relatively low (30%).

An additional finding is that compliance with annual LCS after the initial LCS test is low in the current population. Only 28.7% and 9.9% of eligible patients underwent second and third annual LCS tests, respectively. It is possible that a lack of understanding of the need for annual screening contributes to this attrition. Lack of continuity with primary care providers, limitations in access to care, and other social factors may contribute to this trend as well.

Limitations

The current retrospective chart review examined patients who underwent LCS with LDCT at a single rural intuition in northern Arizona. Although the results are comparable to the NLST, they may not be generalizable to all rural communities across the United States due to the patient population in Kingman, Arizona. The retrospective nature of the study was also limited to data available in the existing hospital electronic medical records. A large number of patients in the current study were lost to follow-up after the initial positive screening with LDCT. The prevalence of malignancy could have been affected by the patients who were lost to follow-up. During the study period, there was also no standardized process for referral to the program, and relied upon outside providers to refer patients for inclusion.

## Conclusions

LCS with LDCT is effective in detecting lung cancer in a rural community at a rate that is similar to that demonstrated in the NLST, with the majority of the cancers being detected at a low stage when they are more likely to be curable. However, the majority of patients with positive LCS tests were lost to follow-up, and the majority of patients who underwent LCS did not return for annual repeat LCS as recommended. Administrative changes could significantly increase the number of cancers diagnosed by LCS by increasing screening compliance with annual LCS after initial LCS, and by increasing compliance to recommendations made when LCS tests are positive. Possible solutions include the use of dedicated LCS program coordinators or patient navigators to communicate eligibility and recommendations to patients and their providers, and/or dedicated tracking software to assist in follow-up reminders and/or additional imaging or procedural recommendations and referrals.
